# Dosimetric evaluation of four‐dimensional dose distributions of CyberKnife and volumetric‐modulated arc radiotherapy in stereotactic body lung radiotherapy

**DOI:** 10.1120/jacmp.v14i4.4229

**Published:** 2013-07-08

**Authors:** Mark K.H. Chan, Dora L.W. Kwong, Gilbert M.L. Law, Eric Tam, Anthony Tong, Venus Lee, Sherry C.Y. Ng

**Affiliations:** ^1^ Department of Clinical Oncology The University of Hong Kong Hong Kong China; ^2^ Department of Clinical Oncology Tuen Mun Hospital Hong Kong China; ^3^ Department of Clinical Oncology Queen Mary Hospital Hong Kong China; ^4^ Theresa Po CyberKnife Center Hong Kong China

**Keywords:** 4D dose calculation, volumetric‐modulated arc radiotherapy, CyberKnife, SBRT

## Abstract

Advanced image‐guided stereotatic body lung radiotherapy techniques using volumetric‐modulated arc radiotherapy (VMAT) with four‐dimensional cone‐beam computed tomography (4D CBCT) and CyberKnife with real‐time target tracking have been clinically implemented by different authors. However, dosimetric comparisons between these techniques are lacking. In this study, 4D CT scans of 14 patients were used to create VMAT and CyberKnife treatment plans using 4D dose calculations. The GTV and the organs at risk (OARs) were defined on the end‐exhale images for CyberKnife planning and were then deformed to the midventilation images (MidV) for VMAT optimization. Direct 4D Monte Carlo dose optimizations were performed for CyberKnife (${4D}_{\text{CK}}$). Four‐dimensional dose calculations were also applied to VMAT plans to generate the 4D dose distributions (${4D}_{\text{VMAT}}$) on the exhale images for direct comparisons with the ${4D}_{\text{CK}}$ plans. ${4D}_{\text{CK}}$ and ${4D}_{\text{VMAT}}$ showed comparable target conformity (1.31±0.13 vs. 1.39±0.24,p=0.05). GTV mean doses were significantly higher with ${4D}_{\text{CK}}$. Statistical differences of dose volume metrics were not observed in the majority of OARs studied, except for esophagus, with ${4D}_{\text{VMAT}}$ yielding marginally higher $D_{1\%}$ than ${4D}_{\text{CK}}$. The normal tissue volumes receiving 80%, 50%, and 30% of the prescription dose ($V_{80\%},V_{50}\%$, and $V_{30\%}$) were higher with ${4D}_{\text{VMAT}}$, whereas ${4D}_{\text{CK}}$ yielded slightly higher $V_{10\%}$ in posterior lesions than ${4D}_{\text{VMAT}}$. VMAT resulted in much less monitor units and therefore greater delivery efficiency than CyberKnife. In general, it was possible to produce dosimetrically acceptable plans with both techniques. The selection of treatment modality should consider the dosimetric results as well as the patient's tolerance of the treatment process specific to the SBRT technique.

PACS numbers: 87.53.Ly, 87.55.km

## I. INTRODUCTION

Stereotactic body radiotherapy (SBRT) is an accepted treatment alternative to surgery for medically inoperable non‐small cell lung carcinoma (NSCLC). Promising results of phase I/II trials in early‐stage I peripherally located lung tumors have led to other studies and clinical trials of locally advanced stage III/IV NSCLC.^1^, ^2^

The delivery technique of SBRT spans a broad spectrum. Using four‐dimensional (4D) dose calculations, Wu et al.^(3)^ suggested that treatment planning with population‐based margins on the free breathing CT resulted in similar mean tumor dose but increased mean lung dose, compared to the individualized internal target volume (ITV) approach, and beam gating at end‐inhale or end‐exhale. For free‐breathing SBRT, an alternative approach to the ITV is the concept of mean target position (MidP). The advantages of planning and delivering the treatment plans at the time‐weighted average tumor position were discussed by several authors.^4^, ^5^ For example, Guckenberger et al.^(5)^ estimated that 2.4 and 6.0 mm margins around the clinical target volume (CTV) at the approximate time‐weighted average position were needed to compensate for motion amplitudes of 10 and 20 mm. This is roughly one‐third of the internal margin. Sonke et al.^(6)^ were among the first to demonstrate the clinical feasibility of using the 4D cone‐beam computed tomography (4D CBCT) to register the soft‐tissue tumor at its time‐weighted average position for treatment verification. Real‐time tumor tracking with CyberKnife is another promising technique to deliver free‐breathing SBRT. This system tracks the implanted surrogates or the soft‐tissue tumor based on a sophisticated feedback loop that measures the respiration signal and the position of the internal fiducial markers/soft tissue tumor and adjusts the treatment beam alignment according to the predicted target position.^7^, ^8^ This advanced motion management technique enables significant reduction of the planning target volume (PTV) margin and potentially better sparing of the normal tissue doses.

Despite the recent development of different advanced radiotherapy delivery strategies, there are limited data quantifying their relative benefits for lung cancers. Many studies have compared the dosimetric advantages of noncoplanar 3D CRT, intensity‐modulated radiotherapy (IMRT) and volumetric‐modulated arc radiotherapy (VMAT).^9^, ^10^, ^11^, ^12^ To our knowledge, there were only a limited number of dosimetric studies comparing ITV‐based 3D CRT/dynamic conformal arc in conventional linac with real‐time tumor tracking using CyberKnife, mainly on the static patient geometry.^13^, ^14^, ^15^ In the lack of further evaluations of the relative advantages of the CyberKnife using target tracking and the VMAT using online 4D CBCT setup verification, we aimed to perform a planning evaluation of these strategies to identify the patient groups that would benefit from the use of one treatment technique or the other.

## II. MATERIALS AND METHODS

### A. Patient data and 4D‐CT imaging

Four‐dimensional computed tomography (4D CT) images of 14 primary non‐small cell lung carcinomas were used to create VMAT plans at the midventilation phase for gantry‐based treatment delivery, and IMRT plans at the end‐exhale phase for CyberKnife. These patients had been treated with either 60 Gy in 3 fractions (n=12) or 48 Gy in 6 fractions (n=2), based on a risk adapted strategy as described by van der Voort van Zyp et al.^(16)^ Tumor characteristics were listed in Table 1. Tumors were considered anteriorly located if the location of their center‐of‐mass were in the area of the anterior half of the ipsilateral lung in the transverse plane. Similar definition applied to posterior tumors. The image thickness was 2.5 mm. Each 4D CT dataset consisted of ten equally time‐binned 3D CT series. All 3D CT series were transferred to MultiPlan treatment planning system (TPS) version 4.0.x (Accuray Inc., Sunnyvale, CA) and Pinnacle^3^ TPS v.9.0 (Philips Medical Systems, Eindhoven, The Netherlands).

**Table 1:** Tumor characteristics

*Patient #*	*Tumor Location*	*GTV (cc)*	${\text{PTV}}_{\text{CK}}(\text{cc})$	${\text{PTV}}_{\text{VMAT}}(\text{cc})$	*TreatmentDose* ^a^ *(Gy)*	*3D Motion (mm)*
1	RUL; attached to anterior chest wall	1.2	6.1	11.2	60	7.6
2	RUL; anteriorly located	73.8	118.7	188.1	60	22.8
3	RUL; anteriorly located	7.5	20.3	34.8	60	4.6
4	RML; anteriorly located	2.5	9.2	16.7	60	8.5
5	RUL; anteriorly located	31.7	63.1	88.1	60	2.1
6	RML; anteriorly located	7.8	22.7	40.8	60	4.6
7	RML; anteriorly located	3.7	12.7	21.0	60	3.7
8	RLL; posteriorly located	0.6	4.2	8.2	60	6.0
9	RLL; posteriorly located	4.2	16.7	27.5	60	9.2
10	LLL; posteriorly located; proximal to esophagus, aorta, and cord	77.9	109.0	130.7	48	4.0
11	RLL; posteriorly located	13.0	31.5	46.9	60	3.6
12	LUL; attached to posterior chest wall and close to esophagus and aorta	15.4	33.4	60.2	48	11.0
13	RUL; posteriorly located	8.1	23.1	32.8	60	7.2
14	RLL; attached to posterior chest wall	10.2	27.8	53.3	60	20.0

a Prescription doses were 60 Gy/3 fractions for peripheral lesions or 48 Gy/6 fractions for central lesions. ${\text{PTV}}_{\text{CK}}=$ planning target volume of CyberKnife plan; ${\text{PTV}}_{\text{VMAT}}=$ planning target volume of 4D VMAT plan.

### B. 4D treatment planning of CyberKnife

For CyberKnife treatment planning, the organ structures were contoured at the end‐exhale in the MultiPlan TPS (4.0.x). A uniform 5 mm safety margin was added to the gross tumor volume (${\text{GTV}}_{\text{CK}}$) to produce the planning target volume (${\text{PTV}}_{\text{CK}}$), as suggested by Lu et al.^(17)^ The primary planning objective was to achieve at least 95% PTV coverage of 100% of the prescription dose. The treatment doses were prescribed to the 62%–83% isodose lines (maximum dose=100%). For critical organs, the dose‐volume limits were listed in Table 2. They were set based on the clinical outcomes reported by van der Voort van Zyp et al.^16^, ^18^ Note that 0.5 cc of selected organs were allowed to get 5% higher dose than the set constraints for the treatment group of 48 Gy/6 fractions.

All treatment plans were created using the built‐in 4D optimization module in the MultiPlan TPS. The 4D optimization is similar to standard three‐dimensional (3D) optimization. It began by a search of a set of candidate beams with optimized weights that best satisfied the user‐specified clinical objectives (e.g., maximize tumor minimum dose, maximize target conformity, and minimize maximum dose of normal organs). It substituted the 3D dose matrix in standard 3D optimization with 4D dose matrices corresponding to independent dose distributions at different breathing states. It further employed B‐spline deformable vector fields that describe the trajectory of each anatomic voxel during respiratory cycle to accumulate doses calculated in different breathing states with equal weight onto a common reference space on which structure contours were defined and evaluated. Beam weights were varied so that the accumulated 4D dose distribution approached the planning objectives. Ideally, the explicit inclusion of organ motion and deformation during the inverse optimization process should ensure 95% PTV coverage of the prescription dose over the breathing cycle. Note that the variations of organ motion pattern and deformation between the treatment fractions were assumed to be negligible, as demonstrated in the previous studies.^19^, ^20^

Since lung consists of a large amount of heterogeneities of low‐density lung tissues, Monte Carlo (MC) dose calculation^(21)^ was applied during the 4D optimizations, as well as in the final dose calculations, forming a direct 4D Monte Carlo optimization framework to ensure proper dose coverage of the PTV and accurate estimates of the doses in the organs at risk (OARs). The sequential algorithm was used for all plan optimizations. The sequential algorithm was executed sequentially as a series of individual optimization steps. Each step performed a linear programming optimization applied to a single objective cost function that was designed to correspond to a specific clinical objective (e.g., target dose coverage, target dose conformity, minimum dose to target, and maximum / mean dose to critical organs). The initial number of beams available to the optimization solution was determined by the number of predefined circular cones. There was no limit to the total number of monitor units (MU) for all plans. But to reduce the overall treatment time, the beam reduction technique was performed to remove from the optimization process those beams whose MU/fraction was below 40 (i.e., corresponding to an average breathing cycle of 4 seconds at fixed dose rate of 600 MU/min) and reoptimized using only the remaining beams. The number of beams of the resulting treatment plans ranged from 100–150(mean=120beams). In addition, the MU/node (i.e., the position of the robot along a certain irradiation path) of each fraction was limited to no more than 200. The final 4D MC optimized dose distribution of the CyberKnife plan was called ${4D}_{\text{CK}}$ dose. To make the MC dose calculation time reasonable, the relative statistical uncertainty was set to 4% during optimizations and reduced to 1% in all final dose calculations. The final dose resolution grid was approximately $1.1\times 1.1\times 2.5\;{\text{mm}}^{3}$.

**Table 2:** Summary of critical organ dose constraints

*Organ*	*Volume*	*Schedule* 3×20Gy *Dose per fraction (Gy)*	*Schedule* 6×8Gy *Dose per fraction (Gy)*
Spinal cord	Any point	8	4.5
Esophagus	Any point	7	6
Heart	Any point	12	8
Trachea and main bronchus	Any point	10	8
Lung	$V_{20}$ (${\text{NTD}}_{2}$)	<31%	<31%

$V_{20}=$ percentage of volume receiving at least 20 Gy normalized to 2 Gy fractions; ${\text{NTD}}_{2}=$ normalized total dose in 2 Gy fractions assuming the α/βratio=3Gy.

### C. 4D treatment planning of VMAT

As mentioned previously, VMAT planning was performed on the MidV images. The midventilation phase was determined by propagating the ${\text{GTV}}_{\text{CK}}$ from the reference exhale phase to other phases in transitive manner by means of constrained intensity‐based deformable image registrations (DIR) that were performed with MiM Maestro v.5.0 (MIM Software Inc., Cleveland, OH). Subsequently, the 3D CT frame in the 4D CT dataset that best approximates the mean target position in the craniocaudal direction was used for VMAT treatment planning, aiming to remove the systematic error from respiratory motion. It is worth noting that choosing a phase image that is not the perfect time percentage corresponding to the MidP induces a generally small baseline shift. However, this discrepancy could be effectively corrected by real‐time tumor tracking and 4D CBCT. In addition, the error of baseline shifts induced by physiological sources generally exceeds the imaging‐induced baseline shift. Nonetheless, minimizing this imaging‐induced shift helps to minimize discrepancies between the positions of surround tissues during treatment delivery.

The GTV‐to‐PTV margin was calculated for each patient according to the van Herk's nonlinear margin formula.^(4)^ The final margin ranged from 6.5 to 9.7 mm in the superior–inferior (SI) direction, 6.2 to 7.4 mm in the anterior–posterior direction (AP), and 6.1 to 7.4 mm in the left–right (LR) direction for the motion amplitude between 1.0 and 22.5 mm (SI), 0.1 and 3.5 mm (AP), and 0.2 to 3.8 mm (LR), respectively.

The SmartArc module in Pinnacle^3^, version 9.0 (Philips Medical Systems, Eindhoven, The Netherlands) was used to create the VMAT plans. Details of the SmartArc algorithm has been described by Bzdusek et al.^(22)^ Briefly, the VMAT optimization began with the generation of coarse segments around the user‐defined arc. This was followed by an intensity modulation optimization on the fluence maps for these segments. The fluence maps were converted to multileaf collimator (MLC) segments, two per angle, and were subsequently filtered and evenly distributed around the arc. Interpolated segments were then added to reach a final fine arc spacing which was set to 4° for all optimized plans. The resulting segments were optimized using direct machine parameter optimization (DMPO) to satisfy the planning objectives and other machine‐specific constraints such as leaf motion, dose rate, and gantry speed.^(22)^

In our study, VMAT plans consisting of one to three full and/or partial arcs were produced for an Elekta Beam Modulator 6 MV linear accelerator (Elekta Oncology Systems Ltd., Crawley, UK) that was equipped with 4 mm MLC and operated at a maximum dose rate of 400 MU/ min. The physical constraints of VMAT planning included a minimum segment area of 4 cm^2^, a minimum segment MU of 20, a 10 iteration segment weight reoptimization to enhance target coverage, and maximum leaf speed of 0.4 cm/gantry degree. The planning objectives were the same to those applied in the CyberKnife planning. The treatment doses were prescribed to the 78%–85% isodose lines. The collapsed cone convolution–superposition algorithm (CCCS) with heterogeneity correction was employed in all final dose calculations at a resolution of $2.5\times 2.5\times 2.5\;{\text{mm}}^{3}$. For each patient, the optimal VMAT plan (${3D}_{\text{VMAT}}$) was applied to recalculate the dose distributions on other 3D CT images in the 4D CT dataset using the same monitor units and the same MLC segments. These individual dose matrices were then warped to the end‐exhale phase by applying the deformation vectors from deformable image registrations on the MiM Maestro workstation. Since the 4D dose distributions of CyberKnife (${4D}_{\text{CK}}$) and VMAT (${4D}_{\text{VMAT}}$) plans were both accumulated to the same breathing phase at end‐exhalation, direct dosimetric comparisons of these dose distributions were feasible using the same set of DICOM RT structure.

### D. Dosimetric evaluations

The 4D dose distributions of CyberKnife and VMAT plans were evaluated by different dosimetric parameters obtained from the dose‐volume histograms (DVHs). First, the conformity of prescription dose to the ${\text{PTV}}_{\text{CK}}$ and the ${\text{PT}}_{\text{VVMAT}}$ was quantified with the new conformity index (nCI), defined as:^(23)^

(1) $$(\text{PI PTV})/{\text{TVIP}}^{2}$$

where *PI* is the volume covered by the prescription isodose line, and *TVIP* is the volume of the PTV covered the prescription isodose line. A larger nCI value indicates less conformity and vice versa. The homogeneity index was excluded from the dosimetric evaluations since dose heterogeneity inside the target volume is generally not a concern in SBRT and even desired. Other evaluation metrics included the mean dose of GTV, the near‐minimum dose of GTV (i.e., dose to 99% of GTV volume ($D_{99\%}$)), the percentage volume of PTV, and organs receiving at least X Gy, $V_{x}$, where X equals 60 or 48 Gy for the evaluation of PTV dose coverage ($V_{60\text{Gy}/48\text{Gy}}$) and 20 Gy for the evaluation of the risk of lung toxicity (i.e., $V_{20\text{Gy}}$), and varies from 80%, 50%, 30%, and 10% of the prescribed doses 60 Gy or 48 Gy (i.e., $V_{80\%},V_{50\%},V_{30\%}$, and $V_{10\%}$) for quantifying the high to low doses in the normal tissue. The near‐maximum dose (i.e., dose to 1% organ volume ($D_{1\%}$)) of the spinal cord, esophagus, and trachea were also compared.

The efficiency of treatment delivery was evaluated by comparing the per‐fraction number of monitor units (MU) and the per‐fraction beam‐on time. The beam‐on time for CyberKnife treatment was estimated by the ratio of per‐fraction monitor units to the dose rate (600 MUs/minute), while it was taken as the estimate from the Pinnacle TPS.

### E. Statistical analysis

Statistical comparisons between CyberKnife and VMAT treatment plans were based on the two‐sided Wilcoxon match‐paired signed rank tests and correlations between variables were performed with Spearman's rank correlation. Differences were considered significant when p<0.05. All statistical analyses were performed with the MATLAB statistical toolbox (The MathWorks, Inc., Nantick, MA).

## III. RESULTS

### A. Tumor dose and target conformity

Dose‐volume indices of the GTV and the PTV for the ${4D}_{\text{CK}}$ and ${4D}_{\text{VMAT}}$ plans, and the original $3\text{D VMAT}({3D}_{\text{VMAT}})$ plans were summarized in Table 3. On average, the organ motion reduced the PTV V60Gy/48Gy by 8.9%, and the ${\text{GTV D}}_{99\%}$ by 2.7%, but hardly changed the GTV mean dose (<0.3%) with and without 4D dose calculations for VMAT plans. By contrast, CyberKnife treatments combining target tracking with 4D inverse planning ensured adequate PTV coverage (95.2%±1.7%) comparing to the ${4D}_{\text{VMAT}}$ plans (86.1%±8.7%). The GTV mean dose was higher for the ${4D}_{\text{CK}}$ plans than the ${4D}_{\text{VMAT}}$ plans by 12.0% in the 60 Gy group and 8.6% in the 48 Gy group, respectively.

**Table 3:** Summary of the dosimetric results for CyberKnife and VMAT plans over the 14 patients

	${4D}_{\text{CK}}$	${4D}_{\text{VMAT}}$	${3D}_{\text{VMAT}}$	*p–value*
*nCI* Mean±1SD	1.31±0.13	1.39±0.24	1.23±0.18	p>0.05 ^a^
Range	1.17–1.64	1.10–1.93	1.06–1.74	p=0.05 ^b^
*GTV Mean Dose Relative To The Prescription Dose (%)* Mean±1SD	133.2±9.5	119.0±4.2	119.4±4.4	p<0.001 ^a^
Range	112.7–145.8	109.6–127.0	109.8–127.8	p<0.001 ^b^
${\text{GTV D}}_{99\%}$ *Relative To The Prescription Dose (%)* Mean±1SD	113.2±9.5	110.3±6.8	113.3±5.4	p>0.05 ^a^
Range	92.7–128.2	96.2–119.3	101.46–20.2	p>0.05 ^b^
$V_{60\text{Gy}/48\text{Gy}}(\%)$ Mean±1SD	95.2±1.7	86.1±8.7	95.0±0.0	p=0.001 ^a^
Range	90.4–97.6	68.4–94.1	94.9–95.1	p>0.05 ^b^
${\text{Lung V}}_{20\text{Gy}}(\%)\;\text{Mean}\pm 1\text{SD}$	6.2±3.7	6.6±3.2	6.3±2.9	p>0.05 ^a^
Range	1.1–14.0	2.5–14.8	2.4–12.7	p>0.05 ^b^
${\text{Cord D}}_{1\%(\text{Gy})}\;\text{Mean}\pm 1\text{SD}$	10.8±7.3	11.7±7.7	12.0±7.8	p>0.05 ^a^
Range	4.3–27.0	2.2–30.7	2.2–30.8	p>0.05 ^b^
${\text{Esophagus D}}_{1\%(\text{Gy})}\;\text{Mean}\pm 1\text{SD}$	9.7±7.1	14.7±11.7	14.8±11.7	p=0.05 ^a^
Range	0.9–24.0	4.7–53.2	4.4–53.1	p=0.05 ^b^
${\text{Trachea D}}_{1\%(\text{Gy})}$ Mean±1SD	9.6±9.9	10.7±10.2	10.5±10.3	p>0.05 ^a^
Range	0.7–33.2	0.2–25.4	0.2–24.5	p>0.05 ^b^

a ${4D}_{\text{CK}}$ vs. ${4D}_{\text{VMAT}}$

b ${4D}_{\text{CK}}$ vs. ${3D}_{\text{VMAT}}$

SD=standard deviation; ${4D}_{\text{CK}}$ and ${4D}_{\text{VMAT}}=4D$ dose distributions of CyberKnife and VMAT plans at end‐exhale phase; ${3D}_{\text{VMAT}}=$ original 3D VMAT dose distributions at midventilation; nCI=new conformity index; $V_{20\text{Gy}}=$ lung volume receiving xx>20Gy; $D_{x\%}=$ dose to X% of organ of interest.

**Figure 1:**
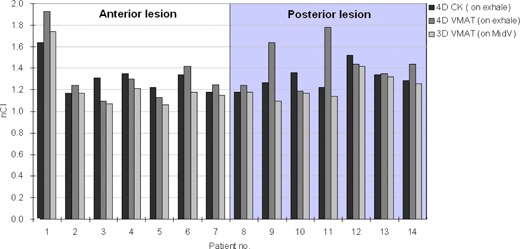
The new conformity index (nCI) are shown for the 4D optimized CyberKnife plans and the 4D accumulated VMAT plans on the exhale images, and the 3D VMAT plans on the midventilation images. The shaded area corresponds to posterior lesions.

The target conformity nCI ranged from 1.17 to 1.64 for the ${4D}_{\text{CK}}$ plans, and from 1.07 to 1.74 for the ${4D}_{\text{VMAT}}$ plans. The organ motion caused observable degradation of the PTV conformity in the ${3D}_{\text{VMAT}}$ plans. The mean nCI increased from 1.23 in the original ${3D}_{\text{VMAT}}$ plans to 1.39 in the ${4D}_{\text{VMAT}}$ plans. The target conformity of the ${3D}_{\text{VMAT}}$ plans was superior to the ${4D}_{\text{CK}}$ plans in six out of seven posterior lesions and in five out of seven anterior lesions (p=0.05), as shown in Fig. 1. The target conformity of the ${4D}_{\text{VMAT}}$ plans was superior to the ${4D}_{\text{CK}}$ plans in only two out of seven posterior lesions and in three out of seven anterior lesions (p>0.05).

### B. Doses of organs at risk

**Figure 2:**
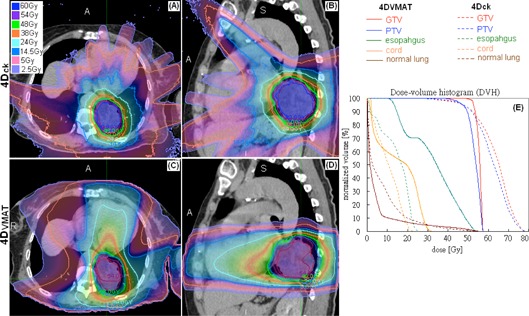
4D dose distributions of the CyberKnife plan (a) and (b), and the VMAT plan (c) and (d)). Dose‐volume histograms (DVHs) of the 4D CyberKnife and the 4D VMAT plans are shown (e) for selected organs. Note the dose levels in ((a)–(d)) roughly correspond to 80%, 50%, 30%, 10%, and 5% of the prescription dose of 48 Gy.

For OARs, the DVH analysis of the 4DCK,4DVMAT' and ${3D}_{\text{VMAT}}$ plans were also given in Table 3. Except for the marginal significance of the difference in $D_{1\%}$ of the esophagus, the dosimetric indices of other OARs were statistically equivalent between ${4D}_{\text{CK}}$ and ${4D}_{\text{VMAT}}$. For normal lung, $V_{20\text{Gy}}$ of ${4D}_{\text{CK}}$ and ${4D}_{\text{VMAT}}$ differed by less than 1% in both 60 Gy group and 48 Gy group. There was notable difference between the ${4D}_{\text{CK}}$ and the ${4D}_{\text{VMAT}}$ dose distributions in one patient (patient no. 10) whose GTV was in close proximity to the esophagus and the cord (Fig. 2). For this patient, the ${4D}_{\text{VMAT}}$ plan using one full arc (180°–179.9°) and one partial arc (300°–179.9°) has not been able to meet the planning objectives for the spinal cord and the esophagus, while the ${4D}_{\text{CK}}$ plan managed to reduce the $D_{1\%}$ of the spinal cord and the esophagus by 40% (21.5 Gy vs. 30.1 Gy) and 117% (24.5 Gy vs. 53.2 Gy), respectively, mainly due to the smaller PTV.

### C. Integral dose and treatment efficiency

**Figure 3:**
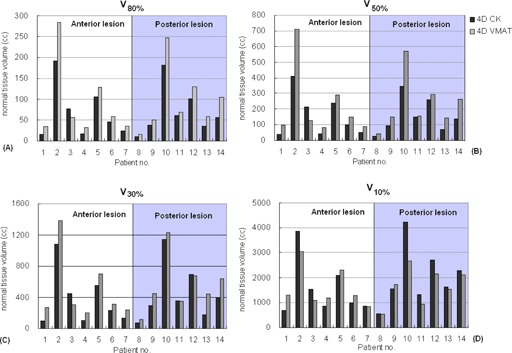
Normal tissue volume receiving $80\%(V_{80\%})$ (a), $50\%(V_{50\%})$ (b), $30\%(V_{30\%})$ (c), and $10\%(V_{10\%})$ (d) of the prescription dose for the 4D optimized CyberKnife (${4D}_{\text{CK}}$) dose distributions (black) and the 4D accumulated VMAT (${4D}_{\text{VMAT}}$) dose distributions (grey). The shaded areas correspond to posterior lesions.

**Figure 4:**
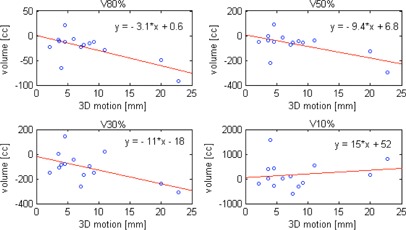
Differences of normal tissue volume receiving at least 80%, 50%, 30%, and 10% of the prescription dose ($V_{80\%}$. $V_{50\%},V_{30\%}$, and $V_{10\%}$) between the 4D CyberKnife and the 4D VMAT plans (${4D}_{\text{CK}}-{4D}_{\text{VMAT}}$) as a function of superior‐inferior tumor motion range. Linear regressions lines (red solid lines) were also plotted.

Comparisons of the high‐ to the low‐dose volumes receiving 80%, 50%, 30%, and 10% of the prescription dose (e.g., $V_{80\%},V_{50\%},V_{30\%}$, and $V_{10\%}$) were shown for each patient in Fig. 3. The ${4D}_{\text{CK}}$ plans significantly reduced the normal tissue from the high‐ to the medium‐dose regions ($V_{80\%}=69\pm 58\;\text{cc}$ and $93\pm 81\;\text{cc},V_{50\%}=156\pm 122\;\text{cc}$ and 226±194cc, and $V_{30\%}=413\pm 348\;\text{cc}$ and 522±379cc in ${4D}_{\text{CK}}$ and ${4D}_{\text{VMAT}}$ plans, respectively; all p‐values <0.05), and yielded comparable low‐dose volumes $V_{10\%}(1793\pm 1141\;\text{cc}$ vs. 1618±742cc; p>0.05). Figure 3 also suggested that the difference of $V_{10\%}$ differed by the tumor location, with CyberKnife producing larger $V_{10\%}$ than VMAT in posterior lesions and vice versa. The differences (${4D}_{\text{CK}}-{4D}_{\text{VMAT}}$) of $V_{80\%},V_{50\%},V_{30\%}$, and $V_{10\%}$ were plotted as a function of tumor 3D motion range in Fig. 4. The Spearman's rank correlation coefficients between the 3D motion and the $V_{x\%}$ indices were −0.58,−0.59,−0.59, and 0.13, respectively. All the coefficients were statistically significant, except for $V_{10\%}$.

The monitor units per fraction and the estimated delivery time were reported for each patient in Table 4. The number of MUs/Gy/fraction ranged from 167 to 357 and from 46 to 68 for CyberKnife treatments and VMAT treatments, respectively. The beam‐on time/fraction ranged from 16.7 to 22.3 minutes for CyberKnife treatments and from 8.3 to 10.8 minutes for VMAT treatments, respectively.

**Table 4:** Summary of the treatment monitor units and the beam‐on time of the CyberKnife and the VMAT plans

	*Monitor Units/Gy/Fraction*		*Beam‐On Time/Fraction (minutes)*	
*Patient No*.	*CyberKnife*	*VMAT*	*CyberKnife*	*VMAT*
1	167	68	16.7	11.2
2	259	58	25.9	11.5
3	172	65	17.2	14.3
4	236	61	23.6	10.9
5	204	60	20.4	12.5
6	205	46	20.5	8.5
7	200	53	20.0	10.8
8	195	46	19.5	8.3
9	357	54	35.7	10.7
10	193	82	15.5	14.3
11	287	55	28.7	9.9
12	271	88	21.7	13.3
13	190	57	19.0	10.7
14	199	54	19.9	9.9
Mean±1SD(20Gy×3)	223±55	56±7	22.3±5.5	10.8±1.6
mean(8Gy×6)	232	85	18.6	13.8
p‐value	p<0.05		p<0.05	

## IV. DISCUSSION

A dosimetric comparison between CyberKnife treatments delivered under the image‐guided real‐time target tracking and VMAT treatments delivered based on the online 4D CBCT setup has been performed using a 4D dose calculation framework to explicitly include the effects of intrafractional organ motion. This study demonstrated that considering VMAT delivery without accounting for the effects of organ motion could lead to biased results that would favor VMAT plans over CyberKnife plans in terms of better target conformity. In a recent study, Atalar et al.^(15)^ evaluated the conformity of linac‐based fixed‐field 3D conformal radiotherapy (3D CRT) and dynamic conformal arc radiotherapy vs. CyberKnife. Without 4D dose calculation, Atalar et al. suggested linac‐based SBRT was associated with superior conformity to CyberKnife. In contrast, Ding et al.^(14)^ showed inferior conformity index (CI) with the 4D accumulated dose distributions of the 3D CRT plans compared to the 3D dose distributions of CyberKnife (1.64±0.29 vs. 1.16±0.06), partly resulting from the inconsistent definition of the PTV (ITV + 5 mm for treatment planning vs. GTV+5mm in calculating the CI). Overall, the organ motion smeared out the dose distributions, causing degraded target conformity and reduced PTV coverage in the VMAT plans designed on the MidV images, although it hardly affected the minimum ($D_{99\%}$) and mean dose of GTV. Using consistent definition of the GTV and the PTV (on exhale phase) for comparisons, we showed that the PTV conformity of the 4D optimized CyberKnife plans was inferior to the 3D VMAT plans, but was comparable to the 4D VMAT plans. This suggests the importance of including the organ motion effects when comparing different treatment strategies. Overall, the CyberKnife plans produced higher GTV mean doses than VMAT plans (Table 1). It is expected that the higher GTV mean doses will not lead to notable differences in clinical outcomes, as several studies have suggested that the local tumor control became saturated beyond biological effective dose (BED) of 100 ${\text{Gy}}_{10}$. The characteristics of the different delivery techniques by VMAT and CyberKnife led to observable differences in the low‐dose volume ($V_{10\%}$) between anterior and posterior lesions, as shown in Fig. 3. The limitation of robotic‐armed linac to irradiate from under the couch attributed to larger difference of $V_{10\%}$ between ${4D}_{\text{CK}}$ and ${4D}_{\text{VMAT}}$ dose distributions in posterior lesions than in anterior lesions (mean=374cc. vs. −22.4cc.). A common characteristic of the ${4D}_{\text{VMAT}}$ plans the greater dose contributions from posterior side, whereas the ${4D}_{\text{CK}}$ plans had more contributions from the superior and inferior sides. For most anterior lesions, the ${4D}_{\text{VMAT}}$ plans resulted in higher dose around the gantry rotation path compared to the ${4D}_{\text{CK}}$ plans whereas, for posterior lesions, the ${4D}_{\text{CK}}$ plans were mostly associated with higher doses anterior to the lesions due to the missing irradiation coming from underneath the patient.

This study used one to three full and/or partial arcs in VMAT planning. It is important to note that the quality of VMAT plan is influenced by the treatment planning system (e.g., RapidArc of Eclipse (Varian Medical Systems, Palo Alto, CA) or SmartArc of Pinnacle) and other factors such as how many arcs are used, and whether coplanar or noncoplanar arcs are used. For example, potential improvement of combining VMAT with static beams has been suggested by Chan et al.,^(24)^ but such hybrid VMAT planning option is not supported in the current version of Pinnacle TPS. In another recent planning study, Merrow et al.^(12)^ compared 3D CRT with VMAT using multiple coplanar and/or noncoplanar arcs. Merrow et al. strongly recommended the use of multiple (partial) arcs to achieve good target conformity and reduction of critical organ doses in lung SBRT. Nonetheless, there were other studies showing that one partial arc can do just as well to meet the dose constraints as multiple arcs in lung SBRT.^9^, ^11^ In this study, we found that one partial or full arc was sufficient for most cases, but it generally required more arcs for the complex cases where GTVs were surrounded by multiple critical organs (patients no. 10 and 12). This is consistent with the early findings of Guckenberger et al.^(20)^ who evaluated the need of more than one single arc for complex‐shaped target volumes. Using the same SmartArc technique as in the present work, they suggested that for simple target shape (prostate), single‐arc VMAT is sufficient while for complex target shape, multiple‐arc VMAT improved dosimetric results compared to the single‐arc VMAT at the cost of increased delivery time, increased MUs, and increased spread of low doses. Furthermore, noncoplanar arc may improve the dose distribution, but it also brings practical problem with respect to setup verification using on‐board 3D/4D CBCT.

Compared with SBRT based on the conventional ITV approach, VMAT treatment using the MidV concept could facilitate significant margin reduction.^(4)^ Over all patients, the ${\text{PTV}}_{\text{VMAT}}$ size was larger than the corresponding ${\text{PTV}}_{\text{CK}}$ by 18.7 cm^3^ because larger margin was needed to compensate for the intra‐ and interfractional variability of organ motion, tumor baseline, and setup. The larger ${\text{PTV}}_{\text{VMAT}}$ did not caused significantly higher $D_{1\%}$ of the spinal cord, esophagus, and trachea or $V_{20\text{Gy}}$ of lung in most cases, except for one patient (patient no. 10). For this patient, 4D VMAT using one full and one partial arc yielded esophagus $D_{1\%}$ of 53.2 Gy/6 fractions, which was definitely unsuitable to undergo SBRT. For the same patient, CyberKnife reduced the esophagus $D_{1\%}$ to 24.5 Gy. The other most notable dosimetric effect of larger ${\text{PTV}}_{\text{VMAT}}$ was the increased normal tissue volume receiving high to medium doses (Fig. 3). A preliminary planning study by Chin et al.^(25)^ has argued that gated SBRT VMAT reduced volumes of irradiated lung volume and some critical organs by irradiating the tumor when it falls into a certain time or amplitude window. However, it is well known that gated beam delivery based on external surrogate is subject to serious uncertainty of the correlation between the internal organ and the external surrogate that is crucial to the geometric accuracy of tumor targeting.^26^, ^27^, ^28^ Korreman et al.^(29)^ also argued that the gating technique does not facilitate margin reduction if there is no image‐guidance technique to ensure tumor at the gated position.

A major advantage of VMAT treatment was the smaller number of MUs and hence much shorter beam‐on time. On average, the VMAT plans consumed 163 MUs/Gy less than the CyberKnife plans did because the beam aperture was opened for most of the VMAT subsegments, whereas CyberKnife made use of small collimated beams to dose paint the target, thus wasting a large amount of MUs. If the VMAT treatments were delivered in the flattening filter‐free mode, the treatment time could be reduced further, down by 20%–350% compared to the conventional VMAT delivery with flattening filter,^30^, ^31^ and thereby decreasing the patient's discomfort and the intrafractional uncertainties. Another downside of CyberKnife is the need of fiducial markers as the tumor surrogate. There were two major problems with fiducial markers: migration and pneumothorax due to the implant process.^(32)^ Most recently, Bibault et al.^(8)^ reported the first clinical outcomes of fiducial free robotic SBRT using the CyberKnife Xsight Lung Tracking System (XLTS). However, XLTS is suitable to a subset of tumors of certain sizes in certain locations. By contrast, 4D CBCT correction protocol has the advantage of direct detection of the soft‐tissue tumor located anywhere in the thorax.

It is important to note that significant uncertainty of our results may result from the use of different deformable image registration algorithms to interpolate the VMAT and the CyberKnife doses on different breathing geometries to the reference end‐exhale frame for dosimetric comparisons. Despite the similarity of these deformable registration algorithms (both are intensity‐based and employ free form deformation), the results of a multiinstitutional study comparing different deformable registration models has demonstrated that differences of the similarity measure, the regularization term, and the optimization method still contributed to uncertainty of the resulting deformation fields.^(33)^ Furthermore, the deformation image registrations applied in this study were based on a simple voxel warping approach without congruent energy and mass mapping.^(34)^ On the other hand, the 4D dose distribution is frequently obtained by deforming the dose calculated on different breathing geometries with equal weight onto a reference geometry. This general approach ignores the dynamic interaction between the MLC's movement and organ movement, or the so‐called interplay effect. Recently, Werner et al.^(35)^ and Rao et al.^(36)^ have separately studied the interplay effect using a plan‐ and patient‐specific beam weight approach for conventionally fractionated step‐and‐shoot IMRT and conventionally fractionated and hypofractionated VMAT. Both groups concluded negligible differences between the general equal‐weight 4D dose calculation approach with the one that specifically considered the relationship between the movements of the MLC and the target. Therefore, one can assume that ignoring the interplay effect does not significantly change our results of the 4D VMAT dose calculations.

Another limitation of the present work and many other dosimetric evaluations was the use of different dose calculation algorithms. Evaluations of dosimetric metrics obtained from different dose calculation algorithms (CCCS for VMAT plans and MC for CyberKnife plans) may subject our results to systematic biases. However, the dose differences between Monte Carlo and collapsed cone convolution–superposition algorithms are generally much smaller than between convolution–superposition and pencil‐beam algorithms.^(37)^ Therefore, results of this study can still be considered more reliable than other previous studies that based their dosimetric comparisons on simple pencil‐beam or convolution–superposition algorithms.^13^, ^14^, ^15^

Several groups have recently developed different frameworks of direct 4D VMAT optimization and have shown promising results.^38^, ^39^ Nonetheless, 4D dose calculation is not a standard option in the majority of commercial TPS. In general clinical setting, this means that the deformable image registration and dose calculation have to be done separately using different tools (e.g., the Pinnacle TPS for dose calculations and MiM Maestro workstation for deformable dose accumulation, as in our case) which requires tremendous manpower and resources. For this study, treatment planning with Pinnacle 810 workstation (2.8 GHz AMD Opteron) and subsequent 4D dose calculation for VMAT plans required at least 3.5 working days (8 hours per working day). For CyberKnife, the built‐in 4D optimization system slightly eased the treatment planning process, but still required at least two working days to obtain a reasonable plan using our planning strategy (higher statistical uncertainty 4% during optimization and 1% in final dose calculation) on the current system (Intel Xeon Processor 3.00 GHz). Whether 4D dose calculation is needed to include the dosimetric effect of organ motion and dynamic beam movement is still controversial. The clinical significance of this dose calculation method should be decided on a patient‐by‐patient basis. Other simplified approaches, such as using the averaged density CT images for dose calculation, should be considered to streamline the treatment planning procedure.

## V. CONCLUSIONS

Both CyberKnife using real‐time tracking and VMAT using online 4D CBCT treatment setup are deemed excellent for lung SBRT as 4D distributions showed comparable target conformity, adequate tumor dose, and dosimetrically acceptable dose‐volume metrics of different critical structures. CyberKnife using image‐guided real‐time tracking may have some advantage over free‐breathing VMAT in the treatment of tumors that show large motion range and/or are surrounded by multiple critical organs, such as those in the central zone, mainly because it can allow smaller safety margin and hence smaller irradiated normal tissue volumes. For peripheral lesions with relatively small motion, the much shorter duration of the VMAT treatment may benefit some patients who have suboptimal physical conditions and cannot tolerate the long duration of the CyberKnife treatment.

## ACKNOWLEDGMENTS

The authors thank Hong Kong Adventist Hospital for the support of this work, and valuable comments of Joyce Leung, Dr. Stewart Tung and Dr. Frank Wong of Tuen Mun Hospital.

## Supporting information

Supplementary Material

Supplementary Material
