# Supplementary figures and images for: Dosimetric evaluation of four‐dimensional dose distributions of CyberKnife and volumetric‐modulated arc radiotherapy in stereotactic body lung radiotherapy
Source: J Appl Clin Med Phys. 2013 Jul 8;14(4):136–49. doi: 10.1120/jacmp.v14i4.4229 (PMC5714543; doi:10.1120/jacmp.v14i4.4229)

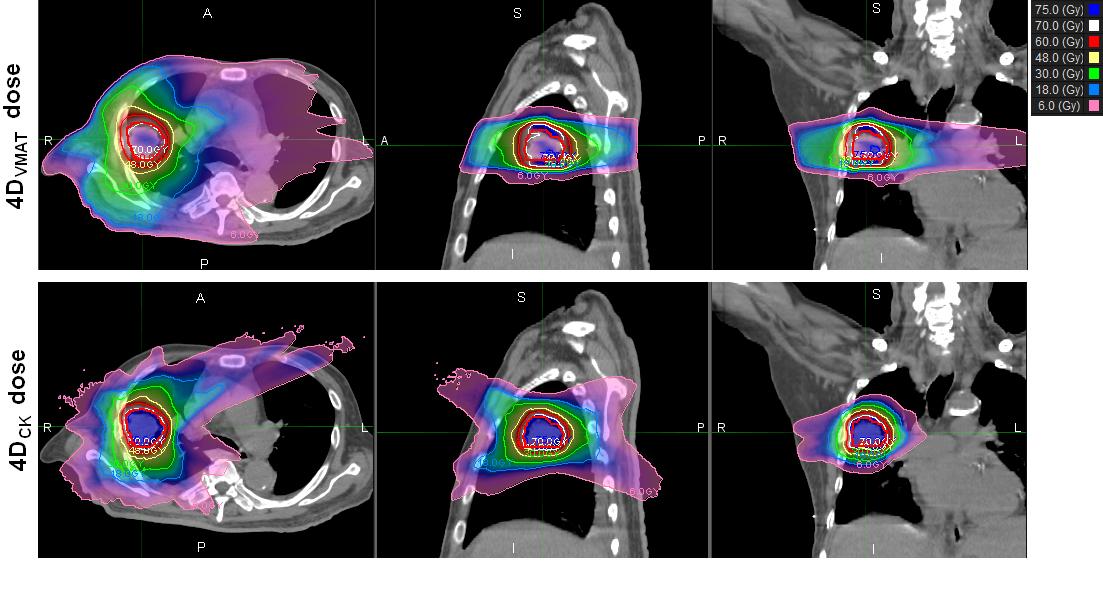

Supplement: Supplementary file 1 — Supplementary Material [file ACM2-14-136-s001.JPG]

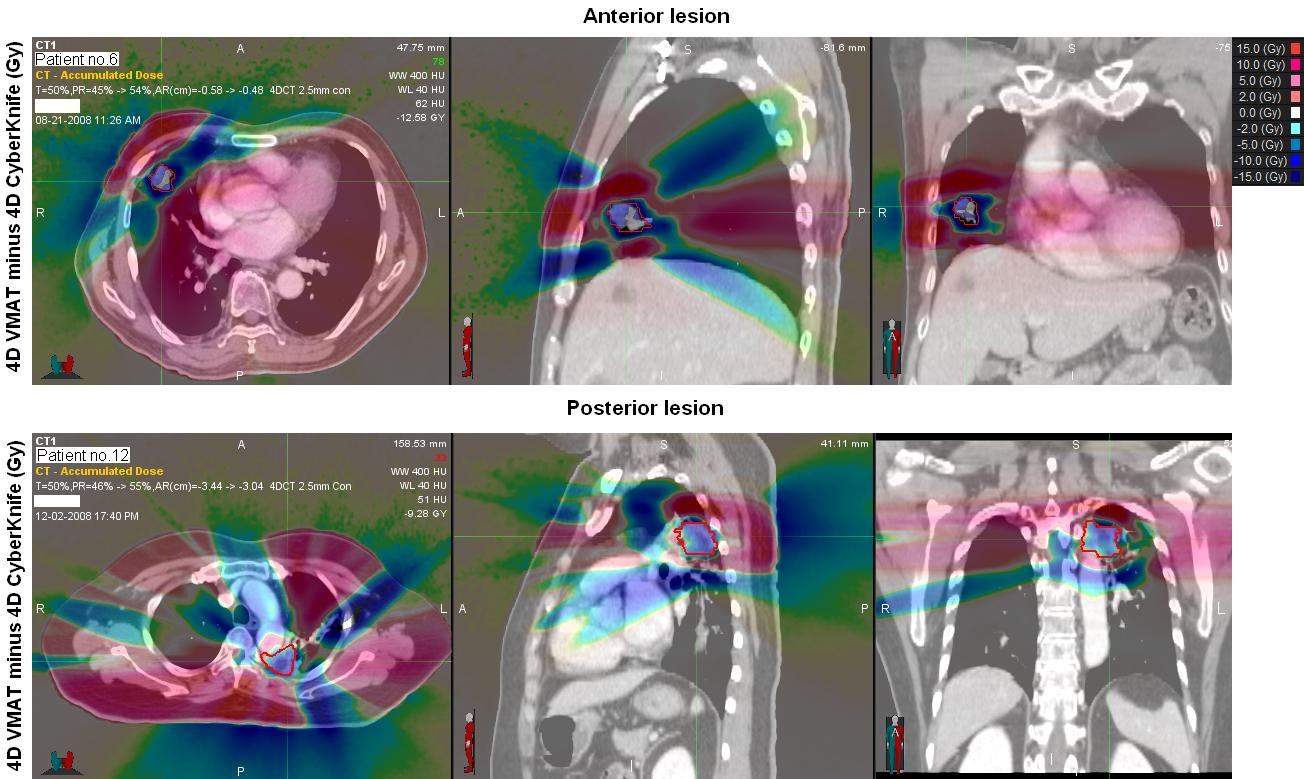

Supplement: Supplementary file 2 — Supplementary Material [file ACM2-14-136-s002.JPG]
